# 

**Published:** 1858-01

**Authors:** 


### TO CORRESPONDENTS.

Interesting communications have been received from Drs. J. N. Graham, J. W. Crooks, Charles Brackett, J. P. DeBruler, C. Goodbrake, P. A. Allaire, and E. Read. They shall appear as fast as our space will permit; and the writers have our warmest thanks for their kindness. A short, but interesting article, came post marked Jacksonville. If the writer will send us his name, we will take great pleasure in publishing it with the article.

